# Preoperative and Intraoperative Localization of Small Pulmonary Nodules for Sublobar Resection: Practical Insights into Percutaneous, Bronchoscopic/Robotic, RFID (SuReFInD), and Hybrid-OR CT Workflows

**DOI:** 10.3390/diseases14060195

**Published:** 2026-05-30

**Authors:** Kanji Tanaka, Masaru Takenaka, Daikichi Meguro, Nobuyuki Take, Teppei Hashimoto, Yasuhiro Fujita, Takehiko Manabe, Katsuma Yoshimatsu, Hiroki Matsumiya, Masataka Mori, Asahi Nagata, Hidetaka Uramoto

**Affiliations:** Second Department of Surgery, School of Medicine, University of Occupational and Environmental Health, Yahatanishi-ku, Kitakyushu 807-8555, Japan; tanaka-kanji@med.uoeh-u.ac.jp (K.T.); teppei3330@med.uoeh-u.ac.jp (T.H.); nasa.med.0204@gmail.com (A.N.); uramoto@med.uoeh-u.ac.jp (H.U.)

**Keywords:** pulmonary nodule, ground-glass opacity, localization, virtual-assisted lung mapping, indocyanine green, robotic bronchoscopy, radiofrequency identification, surgical real-time finger navigation and detection, intraoperative computed tomography

## Abstract

Thin-slice high-resolution computed tomography (CT) has improved the detection of small pulmonary nodules, increasing the demand for minimally invasive diagnostic and therapeutic resection. While lobectomy with lymph node dissection remains the standard surgical approach for many patients with resectable non-small cell lung cancer, accumulating evidence supports sublobar resection for selected small, peripheral, and ground-glass-dominant lesions when sufficient margins are achievable. In thoracoscopic and robotic surgery, localization of nodules ≤10 mm or lesions located >5 mm from the pleural surface can be challenging, and failure to identify the target may lead to conversion, larger resection than intended, or prolonged operative time. Several localization strategies have been developed, including CT-guided percutaneous wire/coil/dye marking, bronchoscopic dye mapping, and virtual-assisted lung mapping (VAL-MAP), robotic-assisted bronchoscopic dye or fiducial localization, radiofrequency identification microtag systems (Surgical Real-Time FInger Navigation and Detection) that provide real-time depth information, and single-stage intraoperative CT-guided marking and resection in hybrid operating rooms. This review synthesizes representative evidence and published outcome ranges, and compares workflows, marker-to-lesion precision metrics, complication profiles, operational burden, and cost structures. We emphasize the practical contrast between two-stage and single-stage workflows, the access-route differences between transthoracic and transbronchial techniques, and the need to report localization-to-incision “time at risk”. We also present an expert-consensus decision algorithm aimed at facilitating tailored selection of localization strategies for modern minimally invasive thoracic surgery.

## Simple Summary

With the widespread use of thin-slice high-resolution computed tomography (HRCT), surgeons increasingly encounter very small pulmonary nodules that are difficult to detect by palpation during minimally invasive surgery. Although lobectomy with systematic lymph node dissection remains the global standard for many resectable non-small cell lung cancers, evidence now supports sublobar resection for selected small, peripheral, and ground-glass-dominant tumors when adequate margins can be secured. However, sublobar resection of tiny or deep lesions often requires reliable intraoperative localization. This review summarizes practical workflows, success ranges, complications, and resource requirements of localization strategies, including CT-guided percutaneous marking, bronchoscopic surface mapping (VAL-MAP and ICG-assisted VAL-MAP), robotic-assisted bronchoscopic localization, bronchoscopic depth-aware RFID tagging (SuReFInD), and intraoperative CT-guided localization in hybrid operating rooms. We compare these approaches using two complementary frameworks—workflow timing (two-stage preoperative vs. single-stage intraoperative localization) and access route (percutaneous vs. transbronchial)—and propose an expert-consensus decision algorithm based on clinical margin needs, access feasibility, time at risk, and institutional resources.

## 1. Introduction

Advances in thin-slice high-resolution computed tomography (HRCT) have improved the detection rate of small pulmonary nodules, including faint ground-glass lesions, leading to an increasing number of patients referred for diagnostic or therapeutic resection [1]. Although lobectomy with systematic lymph node dissection remains the standard surgical procedure for many resectable non-small cell lung cancer (NSCLC) cases [2], contemporary trials and prospective series suggest that sublobar resection can achieve favorable outcomes for selected small, peripheral, and ground-glass–dominant cancers when adequate margins are ensured [3,4]. Consequently, the volume of sublobar resections for tiny lesions is expected to increase.

In minimally invasive surgery, intraoperative localization remains a central challenge for tiny or deep lesions. A frequently cited practical threshold is that nodules ≤10 mm and located ≥5 mm from the pleural surface are often difficult to identify by palpation alone [5]. When a lesion cannot be localized, surgeons may be forced to defer resection, enlarge the extent of resection, or convert to thoracotomy, each of which may reduce the benefit of minimally invasive surgery. To enable precise resection with adequate margins while minimizing loss of lung function, multiple preoperative and intraoperative marking techniques have been developed [6].

## 2. Materials and Methods

### 2.1. Information Sources and Search Strategy

We performed a methods-informed narrative search of major biomedical databases to identify studies on localization and marking techniques for small pulmonary nodules in minimally invasive thoracic surgery. The following sources were searched: MEDLINE (via PubMed), Embase, Scopus, Web of Science Core Collection, and the Cochrane Library. The search window covered 1 January 1976 to 18 February 2026 (last search: 18 February 2026). In addition, we screened reference lists of key articles and performed targeted searches for technique and device names, including virtual-assisted lung mapping (VAL-MAP), indocyanine green–assisted (ICG)-VAL-MAP, radiofrequency identification (RFID), Surgical Real-Time FInger Navigation and Detection (SuReFInD; HOGY MEDICAL CO., LTD., Tokyo, Japan), robotic-assisted bronchoscopy, Monarch (Auris Health, Inc., Redwood City, CA, USA), Ion (Intuitive Surgical Inc., Sunnyvale, CA, USA), shape-sensing bronchoscopy, and for intraoperative imaging workflows (hybrid operating room, cone-beam CT, intraoperative CT). Grey literature relevant to costs and reimbursement was searched in publicly available sources, including government documents and health technology assessments when applicable.

A representative (generic) search concept used across databases was: ((lung OR pulmonary) AND (nodule* OR lesion* OR ground-glass) AND (localization OR marking OR “hook wire” OR microcoil OR dye OR VAL-MAP OR “virtual-assisted lung mapping” OR indocyanine OR ICG OR RFID OR SuReFInD OR “robotic bronchoscopy” OR “robotic-assisted bronchoscopy” OR Monarch OR Ion OR “hybrid operating room” OR “intraoperative CT” OR cone-beam)). Search syntax and controlled vocabulary (MeSH/Emtree) were adapted to each database.

### 2.2. Eligibility Criteria

We included studies and reports that met the following criteria: (i) involved localization/marking of small pulmonary nodules intended for thoracoscopic or robotic wedge resection or segmentectomy; (ii) evaluated or described one or more of the following approaches: CT-guided percutaneous marking (hook-wire, microcoil, dye), bronchoscopic marking (dye/contrast/coil), VAL-MAP (including ICG-assisted variants), robotic-assisted bronchoscopic localization, RFID-based tagging (SuReFInD), or intraoperative CT-guided localization in a hybrid operating room; and (iii) provided clinically relevant details on workflow and/or outcomes (technical success, marker-to-target or mark-to-surface accuracy, intraoperative detectability, conversion or completion of planned resection, time metrics, complications, or operational considerations).

We included randomized trials, prospective/retrospective cohorts, multicenter series, technique papers, and selected case reports when they represented first-in-human milestones or clinically important adverse events. Animal feasibility studies were generally excluded, except when they directly preceded first-in-human application and were necessary to explain the technical concept (e.g., early RFID feasibility).

We excluded conference abstracts without the full text, purely imaging/engineering papers without surgical localization workflow, non-pulmonary indications, pediatric-only series, and duplicate publications reporting the same cohort without additional data.

### 2.3. Study Selection and Prioritization

Given this was a narrative review aimed at practical guidance, study selection was purposive rather than exhaustive. Priority was given to: (i) landmark trials defining standard resection and contemporary evidence supporting sublobar resection in selected patients; (ii) studies that reported quantitative localization performance (success definitions, procedure time, complications); (iii) multicenter or methodologically robust series, when available; (iv) milestone technical reports that introduced widely adopted methods; and (v) authoritative sources describing reimbursement and cost drivers. When multiple publications overlapped in patient cohorts or technique descriptions, we preferentially cited the most complete, most recent, or most widely cited report.

### 2.4. Data Extraction, Operational Definitions, and Synthesis

From each included study we extracted the following data: study design and setting; number of patients/lesions; lesion size and pleural distance, when available; target lesion type (solid vs. ground-glass–dominant); localization workflow (anesthesia, imaging, and sequence of steps); staffing and equipment requirements; time metrics (localization procedure time and, when reported, the localization-to-incision interval or “time at risk”); and complications (pneumothorax, hemorrhage, marker dislodgement/migration, systemic air embolism, infection/fever, and allergic reactions).

For consistency, we used the following practical operational definitions when synthesizing evidence: technical success was defined as successful placement of a marker/tag or creation of a visible/confirmed map and successful intraoperative identification enabling resection of the intended target; clinical success was defined as completion of the planned minimally invasive resection (wedge/segmentectomy) with an oncologically acceptable margin and without unplanned conversion or escalation of resection extent; time at risk was defined as the interval between completion of localization and the start of surgical resection (e.g., skin incision), during which time-dependent failures (dislodgement, dye diffusion, or localization-related symptoms) may manifest. For precision, we preferentially extracted marker-to-target distance or the proportion of markers placed within a prespecified distance (e.g., within 10 mm) when available. When such data were not reported, we used margin-negative planned resection or successful inclusion of the target in the specimen as a pragmatic surrogate. Where studies used different definitions, we reported the authors’ definitions and interpreted findings qualitatively.

Economic and reimbursement information was synthesized using a cost-driver framework separating (i) consumables, (ii) imaging suite time, (iii) operating room and anesthesia time, (iv) personnel time, (v) capital equipment/infrastructure, and (vi) costs of complications and workflow disruptions. Monetary values are presented in the original currency as reported; no currency conversion was performed.

### 2.5. Evidence Limitations

The evidence base for localization techniques is heterogeneous and consists predominantly of single-center observational studies with variable lesion profiles and outcome definitions. Accordingly, we did not perform a formal risk-of-bias assessment or meta-analysis. Instead, we present representative quantitative outcomes, descriptive published ranges, and explicit caveats regarding the definitions used for “technical success”, marker-to-lesion precision, and clinical success. Because several reported precision benchmarks, including the frequently used “within 10 mm” threshold, derive from feasibility or early clinical series, they should not be interpreted as mature multicenter performance standards.

### 2.6. Historical Evolution and Comparative Taxonomy

The development of pulmonary nodule localization reflects a stepwise response to practical surgical problems. Hook-wire localization was first established for nonpalpable breast lesions in 1976 [7] and was later adapted to pulmonary nodules as video-assisted thoracoscopic surgery expanded. Early CT-guided percutaneous hook-wire, dye, lipiodol, and microcoil methods offered simplicity and broad availability but introduced pleural puncture-related risks, dislodgement, and transport-related time at risk [8,9,10,11,12,13,14,15,16,17,18,19,20,21]. Bronchoscopic barium, metallic coil, dye, and VAL-MAP techniques were then developed to avoid pleural puncture and to provide multiple surface landmarks for planned wedge resection or segmentectomy [22,23,24,25,26,27,28,29,30,31,32]. RFID systems evolved from a canine feasibility study in 2014 [33] to the first clinical application in 2020 [34] and a multicenter Japanese evaluation [35], aiming to add real-time depth information. Hybrid operating room workflows and cone-beam or intraoperative CT subsequently addressed the logistical limitations of two-stage localization by permitting confirmation and resection within a single anesthetic episode [36,37]. More recently, robotic-assisted bronchoscopy has moved into clinical use for peripheral lesion navigation and has been extended to dye/fiducial localization workflows [38,39,40].

For practical comparison, we classify the techniques along two axes rather than as isolated devices. First, by access route, localization may be percutaneous/transthoracic (e.g., CT-guided dye or ICG for superficial marks, hook-wire or microcoil for deeper trajectory-based localization), transbronchial/endoscopic (e.g., conventional dye, VAL-MAP, ICG-VAL-MAP, robotic dye or fiducial marking, and depth-aware RFID/SuReFInD tagging), or intraoperative image-guided (hybrid-OR CT/CBCT-assisted marking). Second, by workflow timing, localization may be a conventional two-stage preoperative procedure or a single-stage intraoperative/same-anesthetic procedure. This taxonomy emphasizes clinically relevant trade-offs: surface orientation versus depth-aware guidance, transthoracic complication risk versus transbronchial visibility/reach limitations, and reduced time at risk versus increased anesthesia and operating room utilization.

A standardized precision vocabulary is also needed. For implanted markers, a direct marker-to-target centroid distance or a threshold such as placement within 10 mm is intuitive but is not consistently reported across techniques. For surface mapping methods, the analogous metric may be mark visibility, planned-versus-actual mark location on post-mapping CT, or successful achievement of the intended resection line. For intraoperative CT, the marker-to-lesion relationship can be confirmed immediately, but the outcome may be reported as time at risk or completion of planned resection rather than placement error. Because clinical margin adequacy depends on lesion size, radiographic phenotype, and intended resection type, especially for ground-glass–dominant lesions, margin-negative planned resection should be interpreted as a clinical surrogate rather than a pure localization-precision metric.

## 3. Preoperative Localization Techniques

### 3.1. CT-Guided Percutaneous Marking (Hook-Wire and Related Methods)

Percutaneous CT-guided marking is among the most widely used localization approaches. Following the original hook-wire localization concept developed for nonpalpable breast lesions [7], CT-guided hook-wire placement was adapted for pulmonary nodules and became popular with the expansion of video-assisted thoracoscopic surgery (VATS). In a typical workflow, a planning CT is obtained in breath-hold, the needle trajectory is planned to approach the lesion, local anesthesia is administered, and a hook-wire is deployed into the lung parenchyma near the target. After confirmation CT, the patient is transferred to the operating room for immediate resection.

Reported advantages include relatively simple equipment requirements and low consumable costs. However, percutaneous techniques expose patients and staff to additional radiation and carry procedure-related risks such as pneumothorax, hemorrhage, wire dislodgement, and, rarely, systemic air embolism, which can be detrimental [8,9,10,11,12,13,14,15,16,17,18,19]. Across systematic reviews and technical series, percutaneous localization generally achieves high localization or VATS completion rates, but complication rates vary substantially by marker type, lesion depth, emphysema, puncture route, and waiting time [8,9,10,11,12,13,14,15,16,17,18,19,20,21]. In a representative 2019 single-center Korean hook-wire series (*n* = 113 lesions), localization succeeded in 96.5% of lesions; pneumothorax occurred in 23.0%, hemorrhage in 7.1%, wire dislodgement in 3.5%, and systemic air embolism in 0.8% [20]. The procedure required 23.7 ± 6.3 min on average, and the mean interval between localization and surgery was 34.6 ± 19.9 min [20].

From an implementation standpoint, percutaneous marking typically requires an interventional radiologist, a CT technologist, and nursing support. As such, coordination and transport logistics become a major burden when the CT suite is distant from the operating room. Beyond logistics, the financial viability of localization programs is heavily shaped by local reimbursement and coding rules; in some settings, localization may be bundled into imaging or procedural fees, creating limited direct reimbursement despite additional staffing and infrastructure. For innovative devices, national reimbursement pathways (e.g., application-based categories such as C1/C2 in Japan) may influence adoption and can create a time lag between clinical introduction and stable coverage [41,42,43].

### 3.2. Bronchoscopic Dye Marking and Virtual-Assisted Lung Mapping

Bronchoscopic marking approaches were developed to avoid the risk of systemic air embolism and to expand access to lesions that are unfavorable for percutaneous puncture. Conventional bronchoscopic methods inject dyes (e.g., indigo carmine) or deploy materials (barium, lipiodol, coils) under fluoroscopic or CT guidance to mark the pleural surface near a target lesion [22,23,24,25]. A limitation of conventional bronchoscopic marking is the difficulty of reliably reaching the intended peripheral bronchus, especially for very small airways [26].

**Figure 1:**
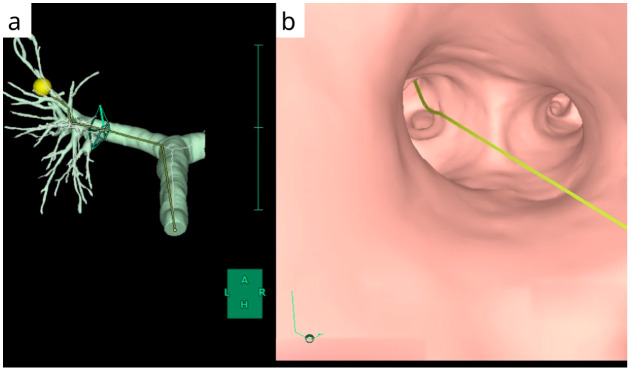
Pre-marking CT-based 3D mapping (VINCENT^®^; FUJIFILM Corporation, Tokyo, Japan). Planned markings were designed on three-dimensional (3D) reconstructions of the bronchial tree (**a**), and bronchoscopy was guided using virtual bronchoscopic navigation (**b**). Source: original figure prepared by the authors from institutional 3D reconstruction/virtual bronchoscopy output; included to illustrate how planned VAL-MAP markings are designed and how virtual bronchoscopy supports bronchoscopic route selection.

VAL-MAP was introduced as a structured bronchoscopic “mapping” strategy. Preoperative thin-slice CT data are used to design multiple planned pleural markings on three-dimensional (3D) reconstructed images (Figure 1). Using virtual bronchoscopy navigation, indigo carmine is injected transbronchially at several sites, followed by a post-mapping CT to confirm the actual marking locations and to reconstruct a map that guides intraoperative resection lines [27,28]. In a representative 2018 single-center Japanese series, the mapping success rate was 90.7% and the median bronchoscopy time was 20.0 min (range 9–90 min), with minor pneumothorax not requiring drainage reported in a small proportion of patients [29]. A multicenter Japanese study of VAL-MAP reproducibility supported the safety of the approach across institutions, although the evidence base remains geographically concentrated in Japan [32].

**Figure 2:**
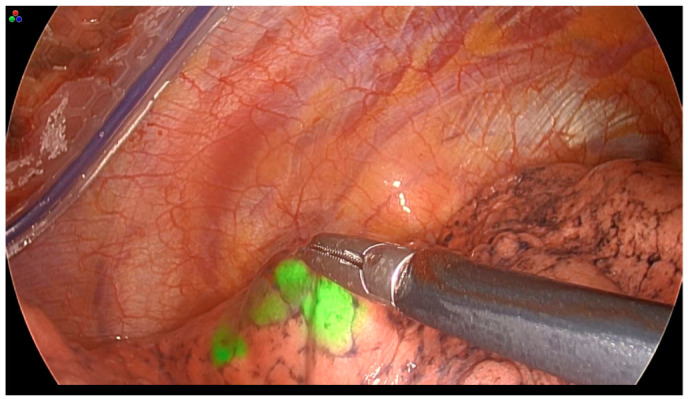
ICG-augmented VAL-MAP under near-infrared imaging. Source: original institutional intraoperative photograph; included to demonstrate near-infrared visibility of ICG-augmented VAL-MAP when surface color contrast is limited.

A key pitfall of dye-only VAL-MAP is limited intraoperative visibility in patients with severe anthracosis or emphysema, and when dye is injected too centrally (i.e., without an adequate subpleural “blush”). In long-term experience summaries, approximately 10% of dye marks may be invisible during surgery because of patient- and technique-related factors [28,30]. To improve visualization, ICG-assisted VAL-MAP (including the ‘dual-staining’ approach) combines indigo carmine with a small dose of ICG, enabling near-infrared thoracoscopic detection even when surface color contrast is poor [31] (Figure 2). Although ICG is generally safe at low doses, allergy risk should be considered.

### 3.3. Robotic-Assisted Bronchoscopic Localization

Robotic-assisted bronchoscopy should be considered a current clinical localization platform rather than only a future direction. Systems such as Monarch and Ion have been introduced into clinical practice for navigation to peripheral pulmonary lesions, and bronchoscopic localization workflows now extend these platforms to dye, ICG, fiducial, or combined dye–fiducial marking [38,39,40]. In a hybrid-OR or same-anesthetic workflow, robotic bronchoscopy can deliver the localization material under fluoroscopic and/or CBCT confirmation and then proceed directly to VATS or RATS, thereby reducing transport and localization-to-incision time at risk [39]. Early localization series remain small and platform-specific: a five-nodule Monarch/CBCT ICG dye-marking report achieved 100% navigation success and 80% dye-localization success [39], while a 2025 pilot series of 10 patients using Monarch with ICG/methylene blue reported successful localization of all nodules with a mean localization time of 16.9 min and no complications [40].

The main advantage of robotic-assisted bronchoscopy is improved peripheral airway stability and reach compared with conventional bronchoscopic tools, particularly when combined with confirmatory imaging. Its limitations include capital and disposable costs, platform availability, CT-to-body divergence, the need for CBCT or other confirmation to reduce mislocalization, and the currently limited evidence base for localization-specific outcomes. Therefore, in this review, robotic bronchoscopic localization is grouped within the transbronchial/endoscopic category and, depending on the marker, may function as either surface mapping (dye/ICG) or depth-aware tagging (fiducial or RFID-compatible workflows).

### 3.4. Bronchoscopic Depth-Aware RFID Tag Localization

**Figure 3:**
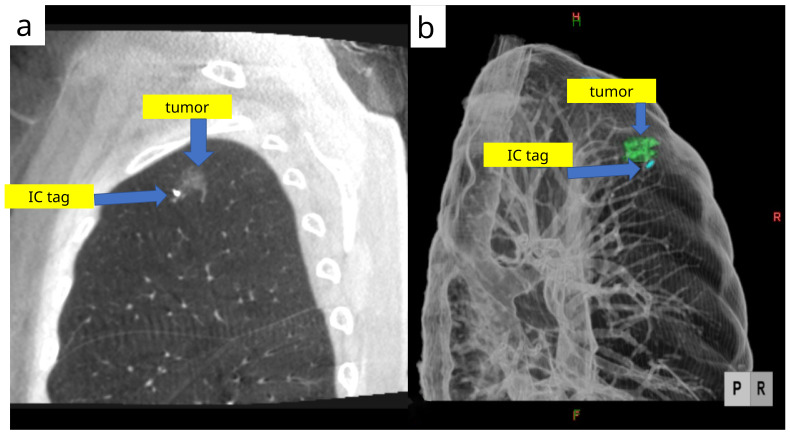
Cone-beam CT images after delivering an RFID (SuReFInD) tag: (**a**) sagittal section; (**b**) 3D reconstructed image. Source: original institutional cone-beam CT image; included to demonstrate postdeployment confirmation of an RFID tag relative to lung anatomy and to clarify the depth-aware nature of this localization strategy.

Surface mapping is effective for many subpleural lesions but provides limited information about depth and deep margin adequacy. RFID systems were developed as a bronchoscopic depth-aware tagging strategy to localize lesions in three dimensions and to support deep margin control without reliance on pleural color changes (Figure 3). The SuReFInD system consists of a bronchoscopic delivery device for a micro-RFID tag with an anchor, a handheld detection probe that provides distance-dependent audio feedback, and a workstation that displays the tag location in relation to 3D lung images [33,34,44]. A canine feasibility study was reported in 2014 [33], and the first human clinical application was published in 2020 [34].

In an early clinical feasibility study of deeply located nodules, markers were placed under general anesthesia using CT-guided bronchoscopy in a hybrid operating theater; 58.3% of markers were placed within 10 mm of the lesion, marker placement required 25.5 ± 14.4 min, and all tumors were resected with negative margins without complications [45]. To expand access beyond institutions with cone-beam CT, later studies evaluated fluoroscopy and virtual bronchoscopic navigation guidance. In a series of 31 patients undergoing segmentectomy, the median bronchoscopic procedure time was 10.0 min, complete placement within 10 mm was achieved in 83.9%, and no pneumothorax or bleeding was observed [46]. A multicenter Japanese study further supported the safety and effectiveness of RFID marking during sublobar resection, with pure ground-glass nodules appearing particularly suitable [35]. However, the current clinical evidence is predominantly Japanese, and generalizability to other healthcare systems, reimbursement structures, and bronchoscopy workflows should be assessed through additional multicenter experience outside Japan.

Practical limitations include device cost, the need for specialized equipment and staff training, and the potential for retained foreign material if a tag is not resected. However, RFID markers are less affected by pleural adhesions, and case reports suggest potential usefulness in revision surgery with extensive adhesions [47].

## 4. Intraoperative Localization in Hybrid Operating Rooms

**Figure 4:**
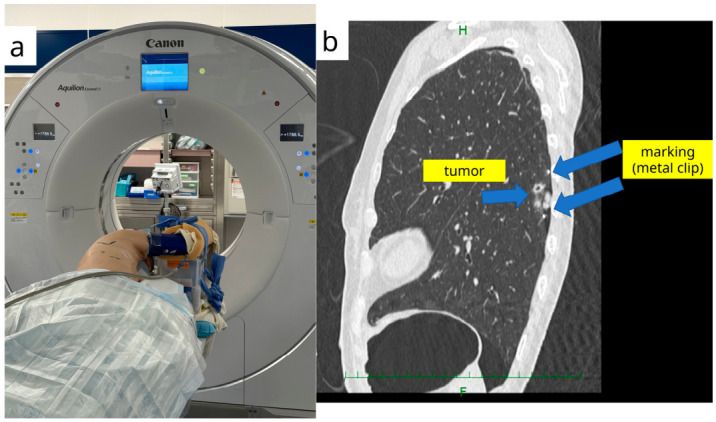
(**a**) Hybrid operating room; (**b**) intraoperative CT after pleural clip marking. Source: original institutional photograph and intraoperative CT image; included to illustrate the single-stage hybrid-OR setup and immediate imaging confirmation after pleural clip marking.

Hybrid operating rooms enable single-stage intraoperative imaging and localization using cone-beam CT (CBCT), multidetector CT (MDCT), or O-arm systems. Under general anesthesia, intraoperative scans are obtained after initial thoracoscopic access, and localization can be achieved using metal clips, dye injection, microcoils, or other markers placed under direct visualization, followed by repeat imaging to confirm the relationship between the marker and target (Figure 4). This approach avoids transport between the CT suite and operating room and can reduce the interval during which complications of localization might occur [36].

In a comparative study, intraoperative CT-guided localization in a hybrid operating room markedly reduced time at risk between completion of localization and skin incision (mean 13.06 min vs. 215.83 min for the conventional two-stage preoperative CT approach), but increased anesthesia time (163.1 vs. 120.61 min) and total operating room utilization time (227.41 vs. 168.68 min) [37]. This finding illustrates the key workflow trade-off: a single-stage approach can minimize time-dependent failure modes, such as marker migration or dye diffusion during transfer, but does so at the cost of longer in-room time and greater dependence on hybrid-OR availability, team training, and radiation-safety protocols.

## 5. Comparative Synthesis and Practical Guidance

Table 1 provides an at-a-glance, practice-oriented comparison of the representative localization techniques discussed in this review. The table contrasts access route, workflow class, information provided, manpower, strengths, limitations, and best-fit indications. This qualitative overview should be interpreted alongside the descriptive outcome ranges in Table 2 and the workflow/economic framework in Table 3.

### 5.1. Reported Outcomes and Complications

Reported success and complication rates vary widely due to heterogeneity in lesion characteristics, definitions (e.g., mapping success, marker-to-target distance, intraoperative visibility, or completion of planned resection), and institutional experience. Table 2 therefore summarizes descriptive ranges and contextual evidence rather than a single point estimate per technique. The table also retains the precision or success endpoint used for each approach and highlights where the marker-to-lesion distance is unavailable.

Global and regional utilization data are sparse. In practical terms, percutaneous CT-guided localization remains the most widely accessible approach because CT suites and hook-wire/microcoil/dye materials are broadly available. VAL-MAP and RFID/SuReFInD have comparatively strong Japanese evidence bases and may be less generalizable to regions without specialized bronchoscopic planning or device reimbursement. Robotic-assisted bronchoscopy and hybrid-OR CT workflows are increasingly used in high-resource centers but are constrained by capital equipment, trained personnel, and scheduling. Because robust annual procedure volumes and institutional penetration rates are not consistently reported across countries, benchmarking should rely on local case volume, complication tracking, time-at-risk measurement, and successful planned-resection rates.

### 5.2. Indications: When to Consider Each Technique

Indications for localization are primarily driven by the probability of failing to identify the lesion during minimally invasive surgery and by the need to secure an oncologically adequate margin. Common triggers include: (i) maximum diameter ≤10 mm; (ii) depth from pleura >5–10 mm; (iii) pure or near-pure ground-glass lesions with limited tactile feedback; (iv) planned sublobar resection requiring a precisely designed resection line; and (v) anticipated pleural adhesions, such as in revision surgery.

Technique selection should follow a hierarchy of clinical need, access feasibility, and institutional workflow. If deep margin control is critical, a depth-aware strategy such as RFID/SuReFInD, fiducial-assisted robotic bronchoscopy, or intraoperative CT confirmation should be prioritized when feasible. If surface orientation and planned cut-line guidance are sufficient, VAL-MAP, ICG-VAL-MAP, or robotic dye marking may be appropriate. If there is no suitable bronchoscopic route or if endoscopic expertise is unavailable, CT-guided percutaneous marking remains reasonable, provided that same-day transfer can be expedited and air embolism risk is considered. If a hybrid OR or same-anesthetic robotic bronchoscopy workflow is available, a single-stage approach may be favored when reducing time at risk is more important than minimizing anesthesia/OR utilization.

### 5.3. Pitfalls and Troubleshooting

Since failures often occur at predictable steps, troubleshooting can be structured around common failure modes and rescue strategies.

In CT-guided percutaneous marking (hook-wire and related), wire dislodgement/migration may be mitigated by minimizing excessive external wire length, expediting transfer to the operating room, and considering alternative markers (microcoil, dye) when transport delay is expected. Pneumothorax/hemorrhage requires post-procedure CT confirmation, readiness for drainage if symptomatic, and avoiding steep needle angles and traversing bullae when possible. Although systemic air embolism is rare, it is severe; prompt recognition of neurologic/cardiac signs and supportive measures such as oxygen administration and patient positioning are essential. Nonetheless, prevention remains challenging; thus, patient selection is critical.

For VAL-MAP/ICG-VAL-MAP, invisible marks caused by anthracosis, emphysema, or central spray can be addressed by increasing the number of marks, employing ICG-assisted near-infrared imaging, verifying mapping through post-procedure CT, and adapting the resection plan accordingly. To manage plan–actual mismatches, incorporating post-mapping CT-based 3D reconstruction facilitates the revision of cut lines and helps to avoid over-reliance on any single mark. For robotic-assisted bronchoscopic localization, CT-to-body divergence and peripheral airway instability should be managed by respiratory control, careful registration, fluoroscopic/CBCT confirmation when available, and readiness to place a fiducial or switch to intraoperative CT or percutaneous localization if dye placement is uncertain. When bronchoscopic reach is limited, considering hybrid operating room or percutaneous alternatives is recommended.

For RFID (SuReFInD), tag misplacement (>10 mm) or wrong bronchus placement should be confirmed with CT after deployment and surgical plans should be prepared for adjustment (e.g., segment selection) or tag removal/redeployment, if feasible. Tag dislodgement can be minimized by cough suppression and careful delivery, with confirmation of tag retention before leaving the bronchoscopy suite. If tag retrieval fails, specimen radiography and systematic counting are needed; if the tag remains in situ, long-term foreign body considerations must be documented clearly.

For intraoperative CT/hybrid operating rooms, workflow delays should be managed by defining a standardized team workflow, including radiation safety checks and imaging protocols to avoid prolonged anesthesia time. Marker shift with ventilation or lung manipulation requires confirmation of the marker–lesion relationship after major lung handling. Repeat scans should also be considered before stapling for critical margins. Radiation exposure can also be minimized by enforcing shielding, distance, and time-minimization principles, while tracking dose where feasible.

### 5.4. Cost Structure, Reimbursement, and Operational Burden

Direct comparisons of absolute costs across localization techniques are difficult because costs depend on local staffing models, imaging access, capital depreciation, reimbursement rules, and whether localization is performed as a two-stage or single-stage workflow. Nevertheless, cost drivers can be organized into a consistent framework: (i) consumables, (ii) imaging time (CT suite or intraoperative CBCT/CT), (iii) anesthesia and operating room time, (iv) personnel time, (v) capital equipment (hybrid OR infrastructure, navigation systems, near-infrared thoracoscopy, robotic bronchoscopy platforms, RFID consoles), and (vi) costs associated with complications and workflow disruptions.

For context, operating room time has been estimated to cost approximately $21–$133 per min (average $62/min) in published hospital analyses, underscoring that even modest changes in operative duration can dominate overall cost differences [48]. A detailed bottom-up analysis in Dutch hospitals estimated costs per min of €9.45 for a conventional operating room and €19.88 for a hybrid operating room, with hybrid operating room costs strongly driven by utilization rates, inventory, and construction costs [49]. Capital costs for building and furnishing a hybrid operating room have been reported to range widely, including estimates of US$1.5–3.6 million [50].

Reimbursement structures can further influence adoption. National health insurance systems differ in whether localization is reimbursed as an independent procedure, bundled into imaging/procedural fees, or covered through device-specific listings. In Japan, reimbursement of novel medical devices that do not fit existing functional categories may require an application-based pathway (e.g., C1/C2), and published analyses highlight potential delays and uncertainty during the listing process [41,42,43]. Similar considerations apply in other jurisdictions with health technology assessment and coding processes.

### 5.5. Proposed Decision Algorithm

**Figure 5:**
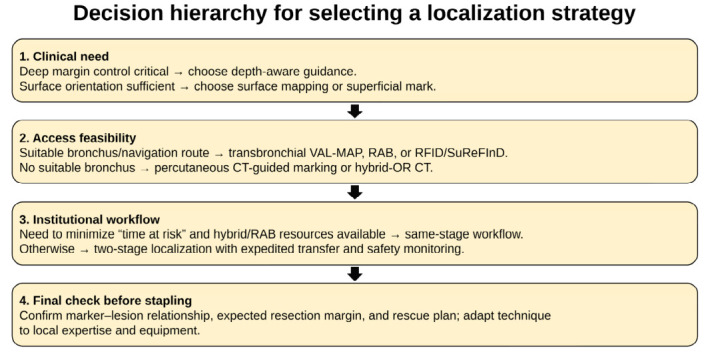
Algorithm for selecting a localization strategy for small pulmonary nodules in minimally invasive sublobar resection. Source: authors’ original synthesis based on the narrative literature reviewed in this manuscript; included to illustrate a practical hierarchy of clinical margin need, access feasibility, and institutional workflow. The algorithm is not evidence-graded and requires prospective validation.

To translate the above comparisons into day-to-day planning, we revised the decision algorithm to start with the clinical need for deep margin control, then access feasibility, and finally institutional workflow constraints (Figure 5). The algorithm is based on narrative evidence synthesis and expert consensus and is intended as a pragmatic starting point rather than a validated guideline.

## 6. Future Directions

The rapid increase in screen-detected small pulmonary nodules is accelerating the need for reliable, scalable, and cost-conscious localization pathways that can support minimally invasive sublobar resection. While current methods are clinically effective, the next phase of development should focus on generating higher-quality comparative evidence, reducing workflow variability, and integrating emerging navigation and imaging technologies into standardized care pathways.

### 6.1. Standardized Reporting and Comparative Evidence

Future studies should adopt harmonized definitions and core outcome sets to enable meaningful comparison across techniques and institutions. At a minimum, reports should describe lesion size, pleural distance, radiographic phenotype (solid vs. GGO-dominant), intended resection type (wedge vs. segmentectomy), achieved margin, conversion or escalation of resection, procedure time metrics (including the localization-to-incision interval or “time at risk”), and complication profiles. Prospective multicenter registries and pragmatic comparative trials—rather than single-center technique series alone—are likely to provide the most actionable evidence for selecting among surface mapping, depth-aware tagging, and intraoperative imaging workflows.

### 6.2. Standardization of Current Robotic Bronchoscopic and Image-Guided Workflows

Because robotic-assisted bronchoscopy is already in clinical use, future work should focus on standardizing localization-specific workflows, confirmation imaging, and comparative outcome reporting. Navigation bronchoscopy, including virtual navigation, electromagnetic navigation, and robotic platforms, is converging with intraoperative imaging (e.g., cone-beam CT in a hybrid OR) to improve placement accuracy while reducing repeated diagnostic CT sessions. Workflows that allow immediate confirmation of marker/tag position, followed by resection in the same anesthetic episode, may reduce time-dependent failures and streamline resource utilization, but they require prospective comparisons that account for anesthesia time, OR utilization, platform cost, and complication avoidance.

### 6.3. Multimodal and Depth-Aware Localization for Margin-Directed Surgery

No single modality addresses all clinical scenarios. A promising direction is the deliberate combination of complementary signals—surface mapping for orientation (e.g., dye/ICG mapping) plus depth-aware markers (e.g., RFID tags or fiducials) for deep margin control—especially for GGO-dominant lesions where palpation is unreliable. As three-dimensional imaging and segmentation tools mature, the field is moving from “finding the lesion” toward “navigating margins”, where intraoperative decision-making can be guided by a continuously updated 3D model of the lesion, planned resection plane, and nearby anatomic constraints.

### 6.4. Radiation Stewardship and Safety Engineering

Because several localization pathways rely on repeated imaging, future work should explicitly incorporate radiation stewardship for both patients and staff. Opportunities include low-dose or ultra-low-dose CT/CBCT protocols, optimizing scan frequency and timing, and workflow designs that minimize repeated repositioning scans. Safety engineering should also address failure modes unique to each technique, such as systemic air embolism risk in percutaneous approaches, dye visibility failure in surface mapping, and retention or migration risk for implanted markers.

### 6.5. Implementation Science: Manpower, Training, and Cost-Effectiveness

Implementation barriers—staffing, scheduling, equipment availability, and learning curves—often determine real-world feasibility more than technical performance alone. Future research should quantify personnel requirements and opportunity costs, evaluate structured training and credentialing pathways, and incorporate cost-effectiveness analyses using transparent cost-driver frameworks (consumables, imaging time, OR/anesthesia time, and capital investment). Establishing reimbursement pathways that reflect true resource use will be essential to avoid inequities in access to advanced localization options.

### 6.6. Toward Personalized Decision Support

Finally, selection algorithms should evolve from expert opinion to data-informed decision support tools. Predictive models that estimate palpability risk and margin difficulty from preoperative CT features, combined with local resource constraints, could help standardize decision-making and reduce unwarranted practice variation. Such tools will require prospective validation and careful evaluation of their impact on clinical outcomes, workflow efficiency, and patient-centered endpoints.

## 7. Conclusions

With increased detection of tiny pulmonary nodules, reliable localization strategies are essential to expand the safety and precision of minimally invasive sublobar resection. CT-guided percutaneous marking remains widely available but carries procedure-related risks and logistical burdens. Bronchoscopic surface mapping (VAL-MAP, ICG-assisted variants, and robotic dye marking) offers a transbronchial alternative that avoids pleural puncture but may provide limited depth information. RFID tagging systems such as SuReFInD provide depth-aware localization and may be particularly useful for deep lesions and revision surgery, albeit with higher device and workflow demands and predominantly Japanese evidence. Intraoperative CT in a hybrid operating room enables a single-stage workflow and minimizes time at risk but increases operating room utilization and depends on capital investment. Therefore, technique selection should consider lesion characteristics, margin strategy, access feasibility, time at risk, and institutional resources, and should be supported by standardized reporting of precision, complications, and operational burden.

## Figures and Tables

**Table 1:** Practical comparison of localization strategies by access route and workflow class.

Aspect	Percutaneous CT-Guided (Hook-Wire/Microcoil/Dye)	Bronchoscopic Surface Mapping (VAL-MAP/ICG-VAL-MAP/RAB Dye)	Bronchoscopic Depth-Aware Tagging (RFID/SuReFInD/Fiducial)	Single-Stage Intraoperative CT (Hybrid OR/CBCT)
Access/workflow	Transthoracic; usually 2-stage; TAR driven by CT-to-OR transfer/scheduling	Transbronchial; usually 2-stage + post-map CT; RAB/hybrid may be same-anesthetic	Transbronchial implanted marker; 2-stage or hybrid; TAR depends on confirmation	Intraoperative image-guided; same-stage; no separate preop marking
Main guidance signal	Surface point/trajectory; indirect depth from needle/wire path	Multiple pleural marks; guides cut line and surface orientation	Real-time 3D proximity/depth signal to tag; supports margin control	Immediate marker-lesion imaging confirmation
Anesthesia/team	Local marking; IR, CT tech, nurse, transfer; GA for resection	Sedation/local bronchoscopy; bronchoscopist + navigation/CT reconstruction; GA for resection	Sedation or GA/hybrid; bronchoscopist + navigation/fluoro + RFID/probe; OR team	GA throughout; hybrid-OR imaging, anesthesia, surgeons, radiation safety
Strengths	Simple, widely available, low consumable cost; useful if no bronchus	Avoids pleural puncture/SAE; multiple marks aid margins	Depth-aware; less dependent on pleural color; useful for deep GGO/adhesions/revision	No transfer; immediate confirmation; low TAR
Limitations	PTX/hemorrhage, rare SAE, dislodgement, radiation/transport burden	Surface-only; invisible marks in anthracosis/emphysema/central spray; post-map CT needed	Cost/training, retained tag risk, confirmation imaging, Japan-heavy evidence	Capital cost, longer GA/OR time, radiation management, limited availability
Best fit	Peripheral lesions; reliable same-day CT-to-OR workflow; limited endoscopic/hybrid resources	Subpleural/surface-oriented lesions; complex wedge/segmentectomy needing surface landmarks	Deep or GGO-dominant lesions needing margin control; revision/intermediate hilar-zone cases	No suitable bronchus; hybrid OR available; minimizing TAR prioritized
Pitfalls/rescue	Wire migration, PTX, transfer delay, SAE; expedite transfer, consider coil/dye	Invisible/misplaced mark; use ICG/post-map CT, avoid reliance on one mark	Tag >10 mm/wrong bronchus/dislodgement; confirm by CT, plan retrieval/redeployment	Positioning/ventilation shift, repeated scans/delays; confirm before stapling

Abbreviations: CBCT, cone-beam computed tomography; CT, computed tomography; GA, general anesthesia; GGO, ground-glass opacity; ICG, indocyanine green; IR, interventional radiology/radiologist; OR, operating room; PTX, pneumothorax; RAB, robotic-assisted bronchoscopy; RFID, radiofrequency identification; SAE, systemic air embolism; SuReFInD, Surgical Real-time FInger Navigation and Detection; TAR, time at risk; VAL-MAP, virtual-assisted lung mapping.

**Table 2:** Descriptive outcome ranges and contextual evidence by access route and localization strategy.

Technique/Evidence Base	Targets + Endpoint	Outcome Range/Key Data	Complications/Time/Caveats
Percutaneous CT-guided hook-wire/microcoil/dye/ICG [8,9,10,11,12,13,14,15,16,17,18,19,20,21] Multi-region single-center series + reviews; most accessible	Small/deep peripheral nodules. Park 2019: Korea, single center; 113 lesions; 10.8 ± 6.1 mm; depth 20.2 ± 12.4 mm. Endpoint: localization/VATS completion or marker stability; marker-centroid distance inconsistently reported [20].	Reviews: high VATS/localization success; hook-wire often mid-90%. Park: 96.5% success [20,21].	PTX/hemorrhage variable; dislodgement uncommon; SAE rare/severe. Park: PTX 23.0%, hemorrhage 7.1%, dislodgement 3.5%, SAE 0.8%; localization 23.7 ± 6.3 min; CT-to-surgery 34.6 ± 19.9 min [20].
VAL-MAP/ICG-VAL-MAP [27,28,29,30,31,32] Mainly Japanese single-/multicenter evidence; ICG improves visibility	Subpleural/surface-oriented lesions; cut-line design. Kuwata 2018: Japan, single center; median tumor 8.0 mm; depth 5.5 mm. Endpoint: mapping success, visible marks, post-map CT confirmation, planned resection/margin [29].	Kuwata: 90.7% mapping success [29]. Long-term: ~10% dye marks invisible; ICG dual staining improves detectability [28,30,31].	Severe events uncommon; minor PTX, pneumomediastinum/alveolar hemorrhage, invisible marks. Bronchoscopy example: 20 min (9–90); safety supported by Japanese multicenter data [29,32].
Robotic-assisted bronchoscopic dye/fiducial [38,39,40] Current clinical platform; early platform-specific localization data	Peripheral small/deep/subsolid lesions reachable bronchoscopically. Chan: Hong Kong, 5 nodules; Yu 2025: China, 10 patients. Endpoint: navigation success, dye/fluorescence visibility, needle-lesion distance, specimen confirmation [39,40].	Chan: 100% navigation, 80% ICG localization [39]. Yu: 10/10 localized; mean 16.9 min; central placement in specimens [40].	No significant marking-related complications in small series. Limits: cost, CT-to-body divergence, need for confirmation imaging, limited comparative data [38,39,40].
RFID/SuReFInD depth-aware tagging [33,34,35,44,45,46,47] Mainly Japanese feasibility, VBN/fluoro, and multicenter data	Deep or GGO-dominant lesions requiring margin control. Yutaka 2022: 11 lesions/12 markers; Komatsu 2024: 31 patients; Miyahara 2023: 182 patients. Endpoint: marker-target distance, within 10 mm placement, margin-negative/planned resection, surgeon utility [35,45,46].	Within 10 mm: 58.3% (early hybrid feasibility) to 83.9% (fluoro+VBN). Cited series achieved negative/sufficient margins; multicenter data support safety and utility [35,45,46].	No PTX/bleeding in cited RFID series; dislodgement 3.2% in Komatsu. Caveats: cost/training, retained tag management, outside-Japan generalizability [46].
Intraoperative CT/hybrid OR [36,37] Single-stage image-guided workflow; comparative timing/resource data	Small/deep solitary nodules. Chao: conventional 2-stage POCT vs. IOCT-guided hybrid OR. Endpoint: immediate marker-lesion confirmation, planned resection, localization-to-incision TAR [37].	No significant outcome differences; IOCT reduced TAR: 13.06 vs. 215.83 min [37].	Transfer-related risk reduced, but GA time longer (163.1 vs. 120.61 min) and OR utilization higher (227.41 vs. 168.68 min); throughput/hybrid-OR availability decisive [37].

Abbreviations: CBCT, cone-beam computed tomography; CT, computed tomography; GA, general anesthesia; GGO, ground-glass opacity; ICG, indocyanine green; IOCT, intraoperative computed tomography; OR, operating room; POCT, preoperative computed tomography; PTX, pneumothorax; RAB, robotic-assisted bronchoscopy; RFID, radiofrequency identification; SAE, systemic air embolism; SuReFInD, Surgical Real-time FInger Navigation and Detection; TAR, time at risk; VAL-MAP, virtual-assisted lung mapping; VATS, video-assisted thoracoscopic surgery; VBN, virtual bronchoscopic navigation.

**Table 3:** Operational and economic considerations by workflow timing and access route.

Dimension	Two-Stage Percutaneous CT	Two-Stage Bronchoscopic Mapping/Tagging	Same-Anesthetic RAB	Single-Stage IOCT/Hybrid OR
Time at risk	Highest with CT-to-OR transfer/scheduling delay; expedite same-day pathway	Bronchoscopy-to-incision + post-map CT/3D reconstruction interval	Reduced if marking + resection in one GA; confirmation imaging adds setup	Lowest reported TAR: 13.06 vs. 215.83 min [37]
Anesthesia/OR	Marking under local; OR mainly resection	Sedation/local bronchoscopy; CT/reconstruction outside OR	Usually GA; robotic platform/team setup	Longer GA and OR utilization: 227.41 vs. 168.68 min [37]
Access-route risk	Pleural puncture: PTX, hemorrhage, rare SAE, dislodgement	Transbronchial: no pleural puncture; limited by airway reach/visibility	Transbronchial with better stability; CT-to-body divergence and cost remain	Intraoperative imaging: radiation/repeat scans, marker shift after lung handling
Cost drivers	CT suite + IR staff; transfer delays; PTX/SAE management	Bronchoscopy team; navigation; post-map CT/3D reconstruction; NIR if ICG	Robotic capital/maintenance; disposables; CBCT/fluoro; OR/GA time	Hybrid suite construction/depreciation; imaging technologist; radiation safety; GA/OR time
Implementation fit	Scalable where CT localization is routine; rapid transfer/emergency protocol needed	Needs bronchoscopic planning expertise; strongest VAL-MAP/RFID evidence in Japan	Best where RAB already established; localization evidence needs validation	Best with high hybrid-OR utilization and standardized team workflow

Abbreviations: CBCT, cone-beam computed tomography; CT, computed tomography; GA, general anesthesia; ICG, indocyanine green; IOCT, intraoperative computed tomography; IR, interventional radiology/radiologist; NIR, near-infrared; OR, operating room; PTX, pneumothorax; RAB, robotic-assisted bronchoscopy; SAE, systemic air embolism; TAR, time at risk; VAL-MAP, virtual-assisted lung mapping.

## Data Availability

No new data were created or analyzed in this study. Data sharing is not applicable to this article. Figures included in this article that were not previously published are provided solely for illustrative purposes, to support the explanation of existing concepts and conclusions derived from the cited literature. These figures do not constitute primary data and were not used to derive or infer any new conclusions.

## Abbreviations

The following abbreviations are used in this manuscript:

CBCT	cone-beam computed tomography
CT	computed tomography
GA	general anesthesia
GGO	ground-glass opacity
HRCT	high-resolution computed tomography
ICG	indocyanine green
IOCT	intraoperative computed tomography
IR	interventional radiology/radiologist
IQR	interquartile range
MDCT	multidetector computed tomography
NIR	near-infrared
NSCLC	non-small cell lung cancer
OR	operating room
POCT	preoperative computed tomography
RAB	robotic-assisted bronchoscopy
RATS	robotic-assisted thoracoscopic surgery
RFID	radiofrequency identification
SuReFInD	Surgical Real-Time FInger Navigation and Detection
VAL-MAP	virtual-assisted lung mapping
VATS	video-assisted thoracoscopic surgery
VBN	virtual bronchoscopic navigation
